# Selective determination of metal chlorocomplexes in saline waters by magnetic ionic liquid–based dispersive liquid–liquid microextraction

**DOI:** 10.1007/s00216-024-05655-5

**Published:** 2024-11-28

**Authors:** Belén Herce-Sesa, José A. López-López, Carlos Moreno

**Affiliations:** Department of Analytical Chemistry, Faculty of Marine and Environmental Sciences, Institute of Marine Research (INMAR), University of Cádiz, 11510 Puerto Real, Cádiz, Spain

**Keywords:** Magnetic ionic liquids, Dispersive liquid–liquid microextraction, Seawater, Metal speciation

## Abstract

In this work, we explore a new dispersive liquid–liquid microextraction (DLLME) method to selectively separate chemical species of Cd and Zn in saline waters. It is based on the use of the magnetic ionic liquid (MIL) methyltrioctylammonium tetrachloroferrate ([N_1,8,8,8_^+^][FeCl_4_^−^]), which allows an efficient and environmentally friendly extraction of the target species. In addition, the paramagnetic component in the MIL simplifies the separation step required in DLLME, allowing for fast separation and recovery of the extracted species with a magnet, without a centrifugation step. The optimum conditions for the separation by MIL-DLLME were 3.3 mg mL^−1^ MIL, sample pH = 8, and an extraction time of 30 min. Under these conditions, metal chlorocomplexes (99.7% and 81.0% of total metal concentration for Cd and Zn, respectively) were quantitatively separated, remaining the free cations in the aqueous samples. In a second step, the extracted metal species were back-extracted with 1 mol L^−1^ HNO_3_ and a re-extraction time of 15 min. For cadmium, this acidic solution separated the neutral complex CdCl_2_ (60.5%), while CdCl^+^ (21.5%) and CdCl_3_^−^ (18.1%) remained in the organic phase. For Zn, the anionic complex ZnCl_3_^−^ (17.3%) was retained by the organic reagent, while ZnCl_2_ (45.7%) and ZnCl^+^ (37.0%) were re-extracted by the nitric acid solution. The separation of the chemical species of metals along the three liquid phases used allowed their quantification in several samples of real seawater and a certified reference material.

**Graphical Abstract:**

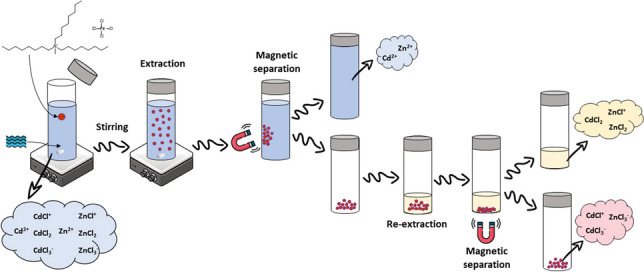

**Supplementary Information:**

The online version contains supplementary material available at 10.1007/s00216-024-05655-5.

## Introduction

Trace metals have attracted much attention in environmental studies due both to their important role in biogeochemical processes and their potential toxicity, even at very low concentration levels. The bioaccumulative and non-biodegradable nature of most of them makes metals very sensitive components of natural waters and thus arouses great interest among researchers. Although some metals, such as Co, Cu, Mn, Ni, and Zn, are essential micronutrients for living organisms, other metals, including Ag, Cd, Pb, and Hg, have been recognized as toxic pollutants and identified as indicators of anthropogenic activities [1–3].

In aquatic ecosystems, trace metals may form different chemical species both with organic and inorganic ligands. Then, to evaluate their environmental behavior, it is not enough to know their total concentrations but also the concentrations of each species, since they can exhibit different properties, i.e., bioavailability, toxicity, etc. [4]. In marine and other saline waters, chloride is the most abundant anion and then, the formation of chlorocomplexes is the predominant process affecting trace metals [5–7]. Some trace elements have a high tendency to form chlorocomplexes (Ag, Cd, Hg, or Pt), while other elements form chlorocomplexes with lower formation constants (Co, Fe, Mn, Ni, Zn) and free hydrated cations are the most abundant species in seawater [8]. On this basis, one metal from each group was selected in this work. Thus, Cd is a toxic element easily complexed in chloride media where it can be found mainly as CdCl^+^, CdCl_2_, and CdCl_3_^−^ [5]. On the other hand, Zn is an essential element that may be also complexed by chlorides but forming weaker complexes and still appearing the free cation Zn^2+^, together with ZnCl^+^, ZnCl_2_, and ZnCl_3_^−^ [6].

In addition to knowing the total concentration of the trace metals, it follows the importance of knowing the chemical speciation of trace metals in natural waters and then, analytical methods to separate, identify, and quantify the different chemical species present in waters are often required. In that case, sample preparation takes an especially important role, given the difficulty of separating and quantifying these chemical species without altering the composition of the sample.

The number of works aimed at the study of metal speciation in water is still small, and they mainly couple a separation procedure with an analytical technique such as atomic absorption spectroscopy (AAS), inductively coupled plasma-atomic emission spectroscopy (ICP-AES), inductively coupled plasma-mass spectrometry (ICP-MS), or an electrochemical technique [9–11].

Although still used, traditional separation methods, such as solvent or solid phase extraction, frequently modify sample conditions, are reagents and time-consuming, and produce big waste amounts [12, 13]. Thus, they are being replaced by more eco-friendly methods, mostly based on microextraction processes, such as liquid phase microextraction (LPME), which can be classified depending on whether the extraction liquid phase contacts directly with the sample or is previously immobilized on a polymeric support. In the first case, the most widespread methods are single-drop microextraction (SDME) and dispersive liquid–liquid microextraction (DLLME), while in the second case, the most extended method is hollow-fiber liquid-phase microextraction (HF-LPME) and its variants [14–16].

Among the advantages of LPME-based methods is the smaller volume of reagents used, which results in a lower waste generation. Nowadays, the greenness or environmental characteristics are a key point in the development of methods for sample preparation. They can be evaluated and quantified using different tools, specifically designed for this purpose with different approaches, and using different variables to quantify the greenness of the method, and thus, the volumes of reagents and waste are combined with other variables such as their toxicity, energy consumption, and safety [17].

Ionic liquids (ILs) are organic salts formed by an organic cation and an organic or inorganic anion, which are liquid at a temperature lower than 100°C [18]. Their unique physico-chemical properties (low vapor pressure, high viscosity, low water solubility, or high chemical stability) make them useful for LPME methods, as an alternative to conventional organic solutions [19]. Besides, the structures of the cation and the anion can be modified by adding functional groups for a specific task and to improve their selectivity towards the metal [20]. ILs have demonstrated their potential for the separation of metal species in waters, even with high chloride concentrations, demonstrating their capacity to selectively extract metallic chlorocomplexes [21–23].

MILs are a subgroup of ILs produced by incorporating a paramagnetic component in their structures, which have a strong response to external magnetic fields, allowing a rapid separation from other phases with a magnet [24]. MIL-based DLLME has been mostly used for extraction of organic compounds, and less frequently for inorganic species such as metals [25]. The use of MILs eliminates the step of centrifugation required in DLLME procedures, simplifying sample preparation and reducing time, energy consumption, and sample handling [26, 27].

This work explores a new sample preparation method for separating and quantifying the chemical species of Cd and Zn present in saline waters by DLLME based on magnetic ionic liquids (MILs).

We have used methyltrioctylammonium tetrachloroferrate ([N_1,8,8,8,8_^+^][FeCl_4_^−^]) to separate the chlorocomplexes of Cd and Zn present in seawater at natural conditions. According to the literature, [FeCl_4_^−^] anion has been selected due to the abundance and low cost of iron materials and their high magnetic properties compared to other anions such as [MnCl_4_^2−^] and [CoCl_4_^2−^] [28]. Likewise, ILs containing [N_1,8,8,8,8_^+^] as a cation present lower solubility in aqueous media than imidazolium ILs, and lower viscosity than phosphonium ILs [10, 29].

## Materials and methods

### Reagents and solutions

Unless otherwise stated, analytical grade reagents were used in this work. Iron(III) chloride hexahydrate (98%), HPLC grade methanol, and ethylenediaminetetraacetic acid (99%) were purchased from Panreac (Barcelona, Spain). Nitric acid (65%) and sodium chloride (99.5%) were provided by Merck (Darmstadt, Germany). Sodium hydroxide (98%) was supplied by Fluka (Buchs, Switzerland), and methyltrioctylammonium chloride was obtained from Alfa Aesar (Kandel, Germany). Atomic absorption spectroscopy standards for Cd and Zn (1000 mg L^−1^) obtained from SCP Science (Quebec, Canada) were used for the calibration curves and to spike the samples during the optimization process. Distilled deionized water (18 MΩ cm resistivity) was obtained with a Millipore Quantum Ultrapure water supplier (Millipore, USA).

The MIL used in this work was synthesized adapting the procedure proposed by Chatzimitakos et al. [30]. Briefly, equimolar amounts of methyltrioctylammonium chloride ([N_1,8,8,8_^+^][Cl^−^]) (2 g, 5 mmol) and iron(III) chloride hexahydrate (FeCl_3_·6H_2_O) (1.34 g, 5 mmol) were dissolved in methanol (20 mL) in a 50-mL round-bottom flask and mixed for 24 h at room temperature. Afterwards, methanol was removed in a rotary evaporator and the obtained product was dried at 60° C overnight. The greenness of the synthesis was estimated by the EcoScale calculator [31].

### Apparatus and instruments

A Laborota 4000 rotary evaporator from Heidolph (Schwabach, Germany) was used during the synthesis of MIL, which was characterized by using a PerkinElmer Lambda XLS UV–VIS spectrometer and a PerkinElmer Spectrum Two FT-IR spectrometer (PerkinElmer, USA).

The pH was measured using a basic 20 pH-meter from Crison Instruments (Barcelona, Spain). Samples and solutions were stirred using a Mix 15 eco magnetic stirrer from 2mag (Munchen, Germany) and an ABT-4 rotary mixer from SBS Instruments (Barcelona, Spain). The concentration of chloride in the samples was measured by a LAQUA PC1100 potentiometric device equipped with a combined electrode from Horiba (Kyoto, Japan).

Metal concentrations in samples and aqueous solutions were quantified using a flame atomic absorption spectrometer (FAAS) model AAnalyst 200 from PerkinElmer (Waltham, MA, USA). For measurement of cadmium (228.80 nm) and zinc (213.86 nm), hollow cathode lamps as a source of radiation as well as a deuterium lamp for background correction were used. The lamp current was fixed at 4 and 25 mA for Cd and Zn, respectively. An acetylene flow of 2.5 L min^−1^ and an air flow of 10 L min^−1^ were employed.

After extraction experiments, MILs were separated by using a S-30–15-N NdFeB magnet from Webcraft GmbH (Gottmadingen, Germany).

### Experimental procedure

**Fig. 1:**
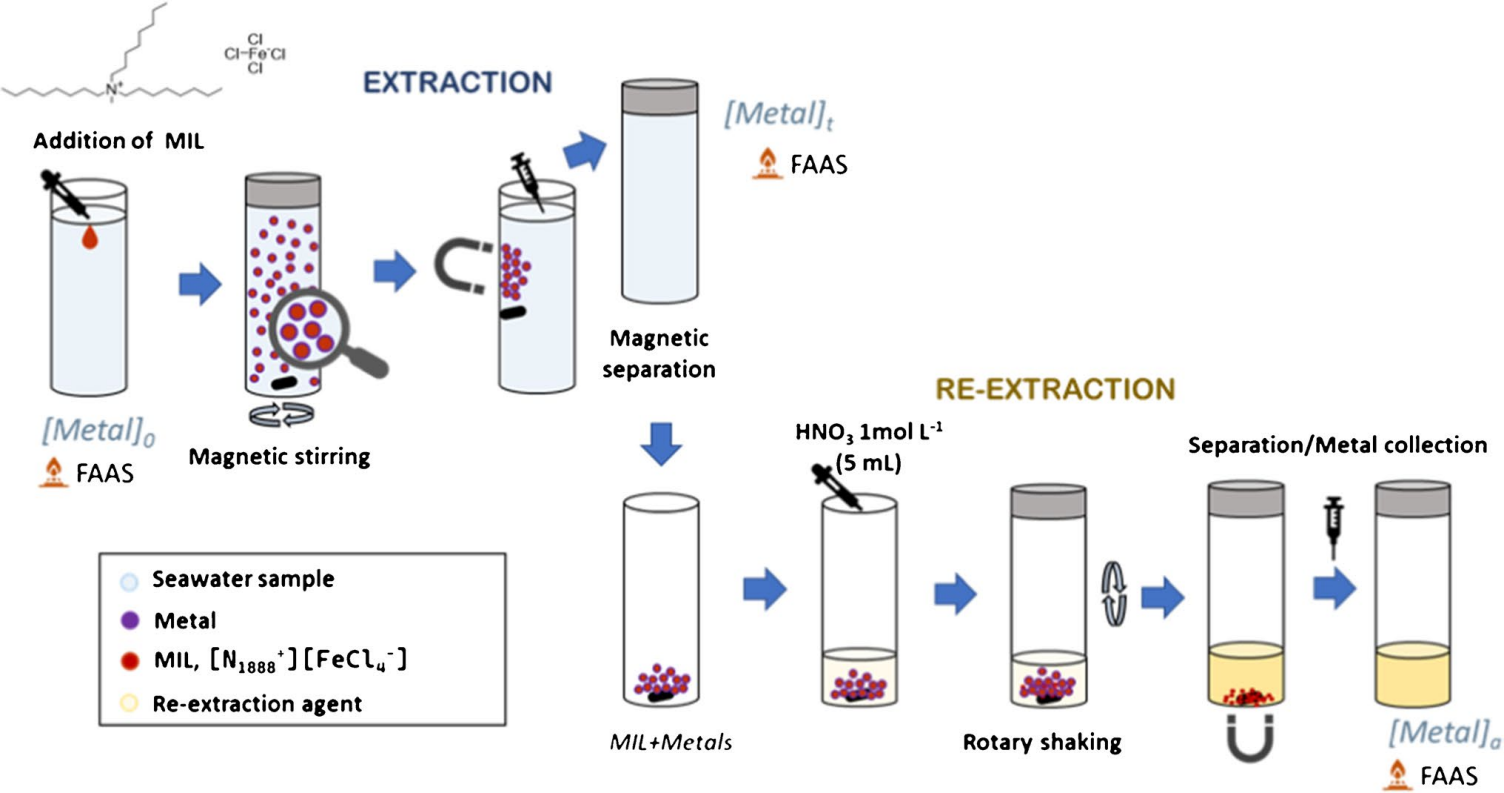
Schematic procedure for MIL-DLLME and back-extraction procedure

The MIL-DLLME procedure is depicted in Fig. 1. For the extraction of the chemical species, a 30-mL water sample was placed in a glass tube together with 3.33 mg mL^−1^ MIL. The mixture was shaken with a magnetic stirrer at 800 rpm for 30 min to favor the contact of the sample with the MIL. After extraction, a magnet was held on the tube’s external wall to attract MIL, and the sample was poured out of the tube. Initial and remaining Cd and Zn concentrations in the samples were measured by AAS, and the extraction efficacies were calculated as follows:

$$\text{Extraction efficacy }\left(\%\right)=\frac{{C}_{\text{i}}-{C}_{\text{f}}}{{C}_{\text{i}}}\bullet 100$$

where *C*_i_ and *C*_f_ are the initial and final concentrations of metals in the sample, respectively. To ensure the stability of the initial metal concentration in the samples, a series of 20 spiked samples was analyzed, obtaining concentrations (*n* = 20) of 1.00 ± 0.10 mg L^−1^ Cd and 0.99 ± 0.05 mg L^−1^ Zn.

After extraction experiments, the sample solution was poured, and MIL loaded with metals (together with the stirring magnet to avoid any loss) remained in the glass tube. Then, 5 mL of 1 mol L^−1^ HNO_3_ was added for the re-extraction of metal species and they were mixed with a rotary mixer to ensure contact with the organic phase, even if adhered to the inner wall of the tube. The efficacy of back-extraction was calculated as follows:

$$Re-\text{extraction efficacy }\left(\%\right)=\frac{{V}_{\text{b}}\bullet {C}_{\text{b}}}{{V}_{\text{s}}\bullet \left({ C}_{\text{i}}-{C}_{\text{f}}\right)}\bullet 100$$

where *V*_s_ is the sample volume, and *V*_b_ and *C*_b_ are the volume and metal concentration in the acidic solution used for back-extraction, respectively. Metal concentrations were measured by AAS.

The described procedure was first used in preliminary experiments to evaluate the potential use of the MIL to separate metal chlorocomplexes from aqueous solutions containing different concentrations of NaCl, up to 0.51 mol L^−1^ at pH 8. Then, optimization experiments were carried out using real seawater collected from the Bay of Cádiz (SW Spain). Samples were collected in low-density polyethylene bottles and filtered using a 0.45-μm pore size nylon filter. After measuring pH and chloride concentration, samples were spiked with 1 mg L^−1^ of Cd and Zn before extraction experiments. After optimization, the method was applied to the separation and determination of chemical species of Cd and Zn in several fortified seawater samples containing different concentrations of Cd (between 0.160 and 1.032 mg mL^−1^) and Zn (between 0.232 and 0.926 mg mL^−1^) and in a certified reference material containing 0.367 mg mL^−1^ Cd and 0.756 mg mL^−1^ Zn (trace metals in seawater—QC3163) from Sigma-Aldrich (Steinheim, Germany).

### Chemical speciation

As a reference of the chemical speciation of metals in water samples with different chloride concentrations, we have used the models published by the International Union of Pure and Applied Chemistry (IUPAC) for Cd [5] and Zn [6]. The models were used to calculate the theoretical concentration of each species in the samples, which were compared with the results obtained with our method. To do this, the samples were extracted using the selected MIL, after which both phases were separated, and the back-extraction process was carried out as described before. In this way, the different metal concentrations in the three liquid phases were established, and the corresponding distribution of the chemical species was calculated.

### Greenness metrics

In recent years, there has been a growing interest in sustainable aspects of analytical methodologies, and today, several tools are available to evaluate them [17]. In this work, we have selected two different methods, which specially focused on the sample preparation steps. Thus, on the one hand, we have used AGREEprep [32], extensively used since its publication, and based on ten categories: in situ sample preparation; safer solvents and reagents; sustainable, reusable, and renewable materials; minimize waste, and products used; sample throughput; automation; energy consumption; post-sample preparation configuration; and safety, each one quantified in a 0–1 scale. This method is dependent on other factors such as the technique used for the quantification of the analytes and then we have also used an alternative method proposed for sample preparation metric of sustainability (SPMS) [33], which does not consider the influence of the analytical instrument.

## Results and discussion

### Synthesis of MIL

The synthesis of [N_1,8,8,8_^+^][FeCl_4_^−^], was performed as described above, and a recovery of 79.2(± 0.5)% was obtained. According to the EcoScale calculator, the synthesis can be considered excellent in terms of economic and ecological performance, with a total score of 79 over 100 points [31]. Table S1 in supplementary material shows the criteria and values used for calculation.

The characterization of the synthetized MIL was done by the VIS and FTIR spectra and following the procedure described elsewhere [30]. Thus, VIS spectrum (Figure S1 in supplementary material) confirmed the presence of the FeCl_4_^−^ anion by the identification of characteristic peaks at 534 nm, 620 nm, 640 nm, and 700 nm. On the other hand, the presence of the cation was confirmed by the comparison of the FTIR spectra (Figure S2 in supplementary material) of the MIL and N_8,8,8,1_^+^ which were practically identical.

### Metal extraction as a function of chloride concentration

**Fig. 2:**
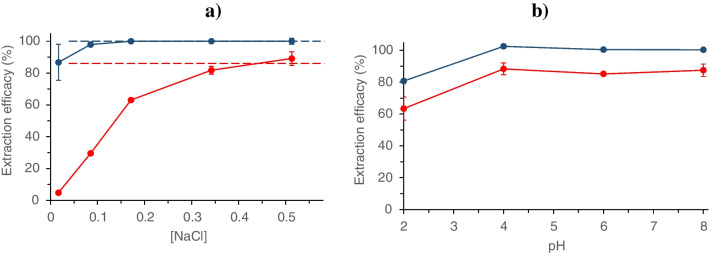
Extraction efficacy of Cd (blue circle) and Zn (red circle) from **a** water samples at pH 8 containing different Cl^−^ concentrations with 3.33 mg mL^−1^ MIL and extraction time of 60 min. Dashed lines indicate the sum of metal complexes (MCl^+^  + MCl_2_ + MCl_3_^−^) at seawater conditions; **b** real seawater as a function of sample pHs. Extraction conditions: 3.33 mg mL^−1^ MIL; extraction time of 60 min. Error bars show standard deviations for *n* = 3

To evaluate the extraction of Cd and Zn at different chloride concentrations, different aqueous solutions containing 1 mg L^−1^ of Cd and Zn at pH 8 were prepared, and different amounts of NaCl were added, between 0 and 0.51 mol L^−1^, to simulate the different conditions of natural waters. The results obtained are shown in Fig. 2a. As can be seen, the extraction was greater for Cd (with a higher capacity to form chlorocomplexes) than for Zn and, although the behavior observed was different for each metal, in both cases the extraction increased with chloride concentration, showing the importance of the chemical species involved and suggesting the preference of the extraction of chlorocomplexes over the free cation. Table 1 shows the distribution of chemical species estimated with the IUPAC’s model for a chloride concentration similar to standard seawater media [5, 6]. The sum of the chlorocomplexes of Cd and Zn was 99.7% and 81.0%, respectively, which is in accordance with the maximum extraction efficacy shown in Fig. 2a, confirming the possibility of separating the metal chlorocomplexes with the selected MIL. Thus, taking into account speciation models and extraction yields, almost quantitative extraction for Cd was achieved, since the metal is mostly present as chlorocomplexes (CdCl^+^, CdCl_2_, and CdCl_3_^−^), while in the case of Zn, all the chlorocomplexes were extracted (ZnCl^+^, ZnCl_2_, and ZnCl_3_^−^), but the free cation Zn^2+^ remained in the aqueous phase.

**Table 1:** Chemical species of Cd and Zn in seawater calculated from IUPAC model

Species	Abundance (%)	Species	Abundance (%)
**Cd**^**2+**^	< 0.01	**Zn**^**2+**^	19.0
**CdCl**^**+**^	21.4	**ZnCl**^**+**^	30.0
**CdCl**_**2**_	60.3	**ZnCl**_**2**_	37.0
**CdCl**_**3**_^−^	18.0	**ZnCl**_**3**_^−^	14.0
**Sum of complexes**	99.7	**Sum of complexes**	81.0

### Optimization of extraction process

The optimization of the extraction conditions was done by using real seawater from the Bay of Cádiz, which was sampled in a location close to our laboratories as described above. For optimization experiments, samples were spiked with 1 mg L^−1^ of Cd and Zn.

#### Effect of sample pH

The results obtained for the extraction of Cd and Zn as a function of sample pH are shown in Fig. 2b. For both metals, the extraction efficacy remained unchanged from pH 4. In a more acidic medium, the protonation of the methyltrioctylammonium cation increases, thus decreasing its affinity for the metal species to be extracted. Given that pH of most natural water samples ranges between 5 and 8, these results ensure the applicability of the method to real samples. Taking into account that the aim of this work was mainly the application of the method to the separation of metal species in seawater samples, pH 8 was used for the next experiments.

#### Effect of MIL concentration

**Fig. 3:**
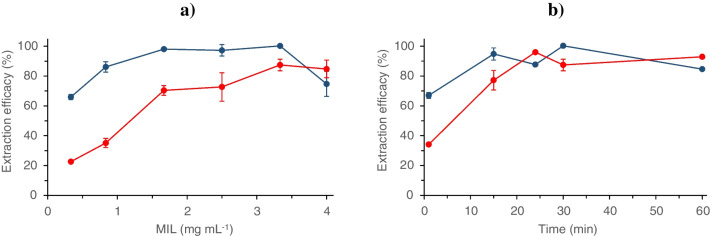
Dependence of extraction efficacy of Cd (blue circle) and Zn (red circle) from real seawater as a function of **a** MIL concentration. Extraction conditions: sample pH = 8; extraction time of 60 min; **b** extraction time. Extraction conditions: 3.33 mg mL^−1^ MIL; sample pH = 8. Error bars show standard deviations for *n* = 3

The concentration of [N_1,8,8,8_^+^][FeCl_4_^−^] as an extraction reagent was varied up to 4 mg mL^−1^. As can be seen in Fig. 3a, an increasing concentration led to an improvement of the extraction of both Cd and Zn up to 3.33 mg mL^−1^ and then, the extraction remained constant or even decreased slightly. Due to their different atomic weights, the molar ratio between the MIL and the two metals is almost double for Cd than for Zn. Because of this, Zn requires a higher concentration of MIL to achieve maximum extraction, and it is this metal that determines the optimal concentration of MIL to be used. To maximize extraction efficacy, 3.33 mg mL^−1^ was selected as the optimum concentration of MIL.

#### Effect of extraction time

Figure 3b shows the results obtained when extraction time was varied within 1–60 min. As observed, extraction of Cd and Zn was measurable in just 1 min but, to obtain optimum extraction efficacy, at least 25–30 min was required. Thus, 30 min was selected as extraction time for further experiments.

#### Extraction and chemical speciation

The extraction mechanisms of metals with ILs are not fully defined, and several approaches have been suggested, mainly based on ion exchange, solvation, and ion pair/neutral extraction [34, 35]. Therefore, ILs can potentially separate both cationic and anionic metal species, as well as uncharged ones.

To calculate the average extraction efficacy of Cd and Zn under optimum conditions (sample pH = 8; 3.33 mg L^−1^ of [N_1,8,8,8_^+^][FeCl_4_^−^]; and extraction time of 30 min), up to ten seawater samples were spiked with different concentrations of Cd and Zn varying between 0.1 and 1 mg L^−1^. They were extracted, and the extraction efficacy of Cd and Zn was 98.6(± 1.0)%, and 82.6(± 3.9)%, respectively. If we compare with the speciation data included in Table 1, we can observe that, for both metals, the extraction efficacy is in good agreement with the sum of chlorocomplexes in each sample estimated by using the IUPAC model, suggesting that these complexes are selectively extracted by the MIL, remaining the free cations in the aqueous sample. The extraction of these free divalent cations would imply the formation of compounds with stoichiometry 1:2, and steric impediments may appear.

### Optimization of re-extraction process

Some preliminary experiments for the re-extraction of metals from the MIL were done by using 1 mol L^−1^ HNO_3_ and 1 mol L^−1^ EDTA. The re-extraction of Cd and Zn with EDTA was negligible, so its use was ruled out, focusing on the study of HNO_3_ as a re-extraction agent, which produced positive results in these preliminary studies. The re-extraction of Cd from MILs with HNO_3_ has been previously described for trihexyl(tetradecyl)phosphonium tetrachloroferrate(III) ([P_6,6,6,14_^+^][FeCl_4_^−^]) [36].

#### Effect of HNO_3_ concentration

**Fig. 4:**
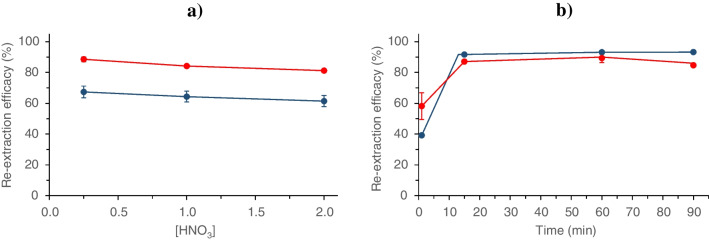
Dependence of re-extraction efficacy of Cd (blue circle) and Zn (red circle) from real seawater as a function of **a** nitric acid concentration. Extraction conditions: 3.33 mg mL^−1^ MIL; sample pH = 8; extraction time of 30 min. Re-extraction conditions: re-extraction time of 30 min; **b** re-extraction time. Extraction conditions: 3.33 mg mL^−1^ MIL; sample pH = 8; extraction time of 30 min. Re-extraction conditions: 1 mol L^−1^ HNO_3_. Error bars show standard deviations for *n* = 3

The concentration of HNO_3_ was varied within the range 0.25–2 M. As can be observed in Fig. 4a, the re-extraction efficacy for both elements varied very slightly throughout the acidity range studied and then, 1 mol L^−1^ HNO_3_ was used henceforth.

#### Effect of back-extraction time

Figure 4b shows the re-extraction efficacy when extraction time was varied between 1 and 90 min. As can be seen, metal extraction increased rapidly during the first 15 min of extraction, and then remained practically constant. Then, the re-extraction time was fixed to 15 min as a compromise value.

To calculate the average re-extraction efficacy of Cd and Zn under optimum conditions (mol L^−1^ HNO_3_ and re-extraction time of 15 min), the same seawater samples previously used to calculate the average extraction efficacy were now re-extracted by using the experimental procedure described above. Thus, the re-extraction efficacy of Cd and Zn was 60.0(± 5.0)%, and 83.0(± 8.3)%, respectively. In Table 2, we show the percentage of the complexes of Cd and Zn extracted in the MIL. If we compare these data with re-extraction efficacies, we can observe that, in the case of Cd, the value of recovery coincides with the abundance of the neutral species CdCl_2_ (60.0% vs. 60.5%) suggesting that nitric acid cannot displace the complexes formed by MIL and CdCl^+^ and CdCl_3_^−^, both species remaining in the organic phase. However, in the case of zinc, the results suggested that nitric acid was able to re-extract ZnCl^+^ and ZnCl_2_ (83.0% vs. 82.7%), remaining ZnCl_3_^−^ unextracted in the organic phase.

**Table 2:** Distribution of the chemical species of Cd and Zn extracted by MIL

Species	Abundance (%)	Species	Abundance (%)
**CdCl**^**+**^	21.5	**ZnCl**^**+**^	37.0
**CdCl**_**2**_	60.5	**ZnCl**_**2**_	45.7
**CdCl**_**3**_^−^	18.1	**ZnCl**_**3**_^−^	17.3

Summarizing, in the case of Cd, the free cation Cd^2+^ remains in the water sample, CdCl^+^ and CdCl_3_^−^ stay in the organic phase, and CdCl_2_ is separated to the acidic aqueous solution. On the other hand, Zn^2+^ also remains in the water sample, ZnCl_3_^−^ is retained in the organic phase, and ZnCl^+^ and ZnCl_2_ are separated to the acidic aqueous solution used for the back-extraction.

### Analytical parameters and application

As in any analytical method based on a previous separation/preconcentration step, the limit of detection depends not only on this sample treatment, but also it depends strongly on the analytical technique used. In this case, under optimum conditions and by using F-AAS for quantification, the limits of detection obtained for total metal concentration were 0.021 mg L^−1^ for Cd and 0.035 mg L^−1^ for Zn.

The new method was applied to the determination of the chemical species of Cd and Zn present in four samples of real seawater fortified with different concentrations of both metal between 0.1 and 1 mg L^−1^ and to a certified reference material of seawater containing 0.367 mg L^−1^ Cd and 0.756 mg L^−1^ Zn. The results obtained are shown in Table 3. As can be observed, all the results obtained were in good agreement with those used as reference, calculated by using the IUPAC model previously introduced. The precision of the results was evaluated by the corresponding RSD, the average value being 5.9%. In addition, the absence of bias in the results was confirmed by using a *t*-test. Experimental values of *t* were always less than the critical value *t* = *4.30* (*p* = 0.05, *n* = 3); thus, the null hypothesis was retained and there was no significant difference between known and measured concentrations.

**Table 3:** Concentration of chemical species in seawater samples

Sample^a^	Cd						Zn					
	Theoretical speciation^b^			Experimental results^c^			Theoretical speciation^b^			Experimental results^c^		
	Cd^2+^	CdCl_2_	CdCl^+^/CdCl_3_^−^	Cd^2+^	CdCl_2_	CdCl^+^/CdCl_3_^−^	Zn^2+^	ZnCl^+^/ZnCl_2_	ZnCl_3_^−^	Zn^2+^	ZnCl^+^/ZnCl_2_	ZnCl_3_^−^
1	0.005	0.096	0.063	< 0.021	0.090 ± 0.017 (− 6.8%)	0.060 ± 0.012 (− 4.7%)	0.044	0.155	0.032	0.047 ± 0.014 (− 8.2%)	0.165 ± 0.003 (6.1%)	0.034 ± 0.001 (4.1%)
2	0.008	0.157	0.102	< 0.021	0.139 ± 0.010 (− 11.2%)	0.093 ± 0.007 (− 9.2%)	0.061	0.216	0.045	0.053 ± 0.005 (− 8.2%)	0.232 ± 0.004 (7.3%)	0.048 ± 0.001 (5.3%)
3	0.011	0.223	0.146	< 0.021	0.211 ± 0.018 (− 5.3%)	0.141 ± 0.012 (− 3.2%)	0.089	0.314	0.066	0.082 ± 0.009 (− 8.2%)	0.297 ± 0.009 (− 5.6%)	0.061 ± 0.002 (− 7.4%)
4	0.031	0.622	0.407	< 0.021	0.647 ± 0.033 (4.0%)	0.433 ± 0.022 (6.4%)	0.176	0.620	0.130	0.162 ± 0.012 (− 8.2%)	0.637 ± 0.012 (2.6%)	0.131 ± 0.002 (0.7%)
CRM	0.011	0.221	0.145	< 0.021	0.230 ± 0.011 (3.7%)	0.153 ± 0.007 (6.0%)	0.144	0.507	0.106	0.132 ± 0.023 (− 8.2%)	0.493 ± 0.023 (− 2.7%)	0.101 ± 0.005 (− 4.5%)

^a^Samples of seawater fortified with total concentration (in mg L^−1^) of Cd/Zn as follows: (1): 0.160/0.232; (2): 0.260/0.323; (3): 0.370/0.469; (4): 1.032/0.926; CRM (QC3163): certified reference material containing 0.367/0.756

^b^Calculated from IUPAC’s model (Powel et al., 2011; Powell et al. 2013)

^c^This work. Results expressed as average concentration (in mg L^−1^) ± standard deviation (*n* = 3) and the corresponding relative error (in brackets). Concentration of Cd^2+^ expressed as below limit of detection

#### Greenness assessment

The greenness of the new method was evaluated by using two methods: AGREEprep and SPMS. Table S2 shows the criteria and values used for both methods.

As explained before, AGREEprep is based on ten variables related to the sustainability of the analytical method, which are quantified in a 0–1 numerical scale and translated into a graphical representation using colors (a red–orange-green scale). This tool brings clarity and merges quantitative and qualitative aspects, although it also has some drawbacks, such as a strong influence of the instrumental technique used [32].

SPMS focuses exclusively on sample preparation steps, which are evaluated in a numerical 0–10 scale and represented in a clock-like pictogram with colored squares, and tries to reduce some of the drawbacks of previous tools [33].

**Fig. 5:**
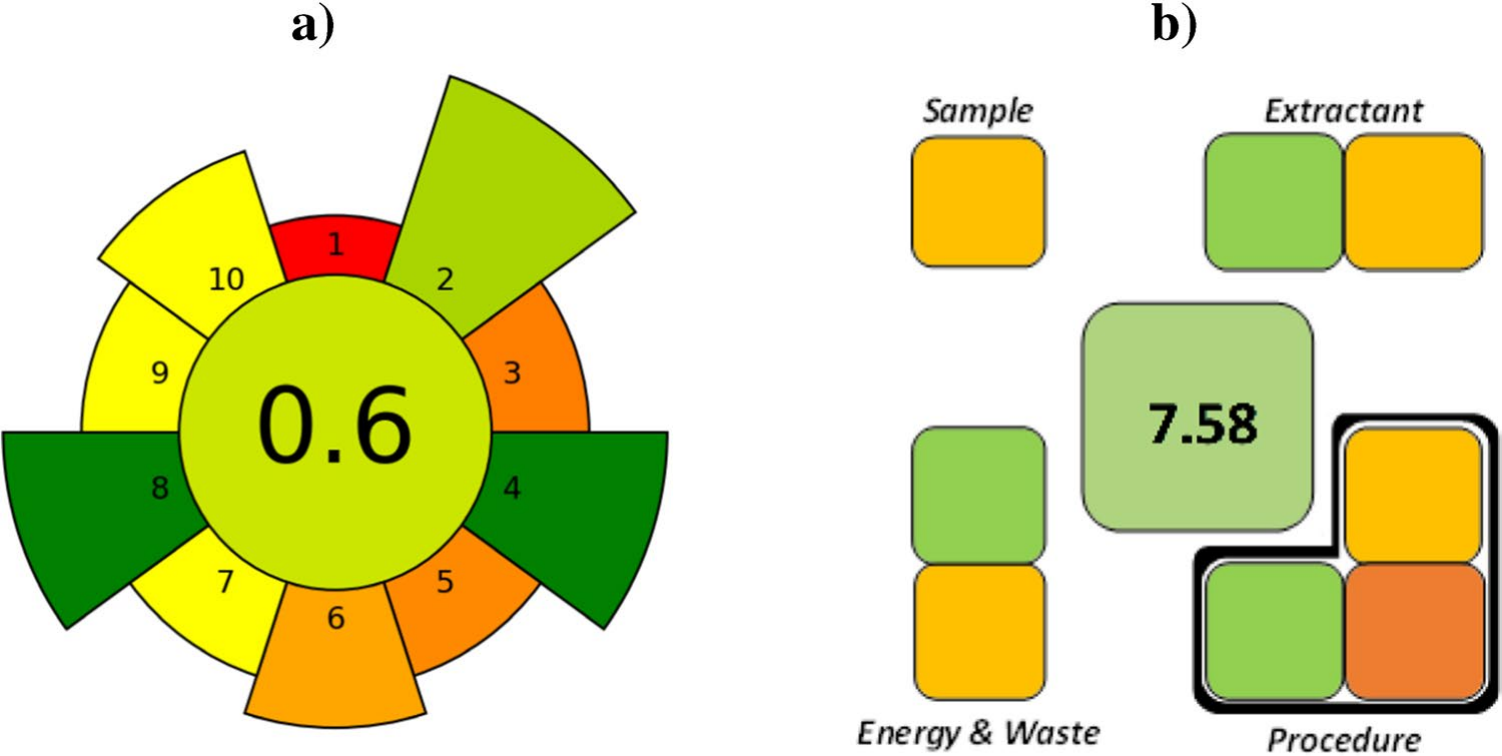
Pictograms obtained with AGREEprep (**a**) and SPMS (**b**)

The corresponding pictograms obtained in the present work are shown in Fig. 5. As can be seen, both tools identify the proposed method as a sustainable method, with scores of 0.61/1 (AGREEprep) and 7.58/10 (SPMS). The best metric obtained with SPMS may be related to the greater specialization of the tool in the sample preparation stage, where the greatest innovation of the new method is focused. However, it should be noted that one of the advantages of the new method is the use of a very small amount of MIL, a variable that is specifically taken into consideration by AGREEprep but not by SPMS.

## Conclusions

The proposed DLLME method based on [N_1,8,8,8_^+^][FeCl_4_^−^] provided an easy separation of metal species from seawater and other saline waters, without altering natural sample conditions, and by using the magnetic properties of the reagent to easily separate both phases with a magnet, eliminating the centrifugation step. The species are separated along the three liquid phases involved: the water sample, the organic phase containing [N_1,8,8,8_^+^][FeCl_4_^−^], and the aqueous acidic solution. After the extraction step, both free cations (Cd^2+^ and Zn^2+^) remain in the water sample, while all the chlorocomplexes are extracted to the organic phase. Then, the different affinity of the MIL for metal species causes some differences in the distribution of the complexes, and after re-extraction with nitric acid, the organic phase retains CdCl^+^ and CdCl_3_^−^ for Cd and only ZnCl_3_^−^ for Zn. Finally, the acidic aqueous solution used for back-extraction separates CdCl_2_ for Cd, and ZnCl^+^ and ZnCl_2_ for Zn.

The performance of the method gives it great interest for environmental studies in general and for marine studies in particular.

## Supplementary Information

Below is the link to the electronic supplementary material.

Supplementary file1 (DOCX 673 KB)
