# Supplementary material for: Selective determination of metal chlorocomplexes in saline waters by magnetic ionic liquid–based dispersive liquid–liquid microextraction
Source: Anal Bioanal Chem. 2024 Nov 28;417(7):1369–79. doi: 10.1007/s00216-024-05655-5 (PMC11861130; doi:10.1007/s00216-024-05655-5)
Supplement: Supplementary file 1 — Supplementary file1 (DOCX 673 KB) [file 216_2024_5655_MOESM1_ESM.docx]

Table S1. Criteria and values used in Ecoscale calculator

| **ECOSCALE** | |
| --- | --- |
| **Reagents** | |
| C_25_H_54_NCl | 0.367 mmol |
| Iron(III) chloride | 0.367 mmol |
| CH_4_O | 246.877 mmol |
| **Conditions** | |
| Yield | 79.2% |
| Technical Conditions | Common set-up |
| Temperature/Time | Room temperature, <1h |
| Workup and purification | Distillation |

Table S2. Criteria and values used for greenness evaluation with AGREEprep and SPMS methods

| **AGREEprep** | |
| --- | --- |
| **Criterion** | **Value** |
| Sample preparation placement | Ex-situ |
| Hazardous materials | 0.1g |
| Sustainability, renewability and reusability of materials | 25-50% |
| Waste | 0.1g |
| Size economy of the sample | 15mL |
| Sample throughput | 2.66 |
| Integration and automation | 2 steps/semi-automated |
| Energy consumption | 10 |
| Post-sample preparation configuration for analysis | AAS |
| Operator’s safety | 2 hazards |

| **SPMS** | |
| --- | --- |
| **Sample amount** | |
| Sample amount (mL or g) | 10 < x ≤ 50 |
| **Extractant information** | |
| Amount of extractant (mL or g) | ≤ 0.1 |
| Nature of extractant | Alternative degradable |
| **Procedure information** | |
| Number of steps | 2 < x ≤ 4 |
| Extraction time (min) | 15 < x ≤ 60 |
| Additional steps after extraction | No additional steps |
| Samples throughput | Multiple samples |
| **Energy consumption** | |
| Dispersion/stir | Stir plate |
| Separation | No centrifuge |
| Temperature | Room temperature |
| **Total waste** | |
| Waste (mL or g) | ≤ 10 |
| **Reusability of extractant** | |
| Reusable | No |

Figure S1. Visible absorption spectrum of [N_1,8,8,8_^+^][FeCl_4_^-^]. Arrows indicate peaks at 534nm, 620nm, 640nm and 700nm.

Figure S2. FTIR spectra of methyltrioctylammonium chloride ([N_1,8,8,8_^+^][Cl^-^]) (A) and methyltrioctylammonium tetrachloroferrate ([N_1,8,8,8_^+^][FeCl_4_^-^]) (B)


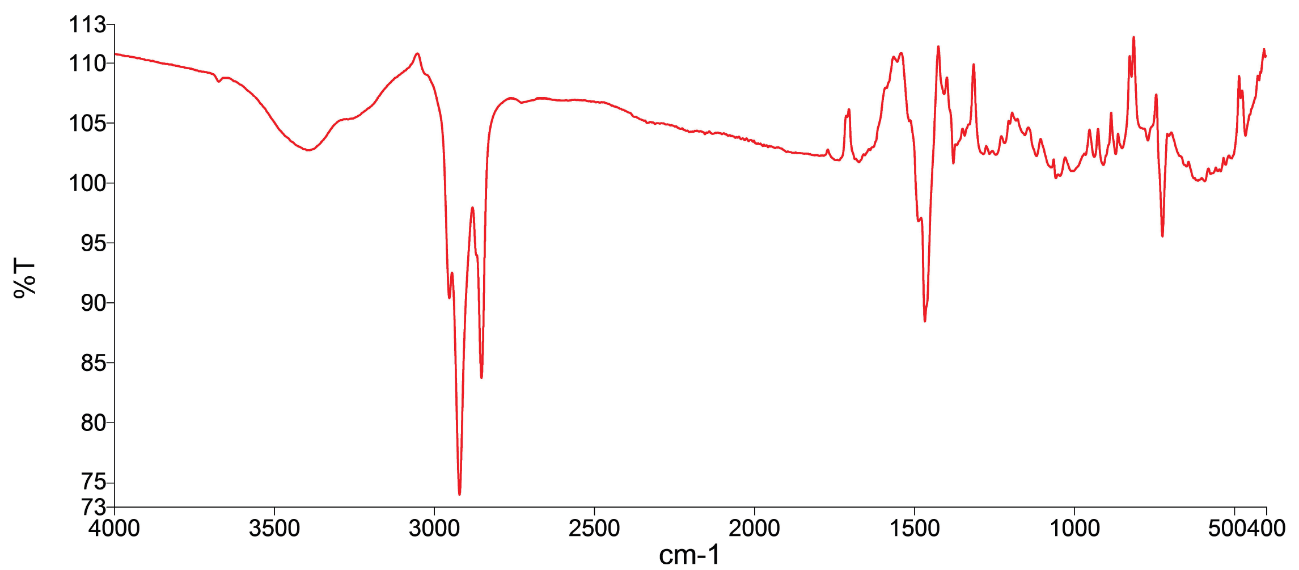


A


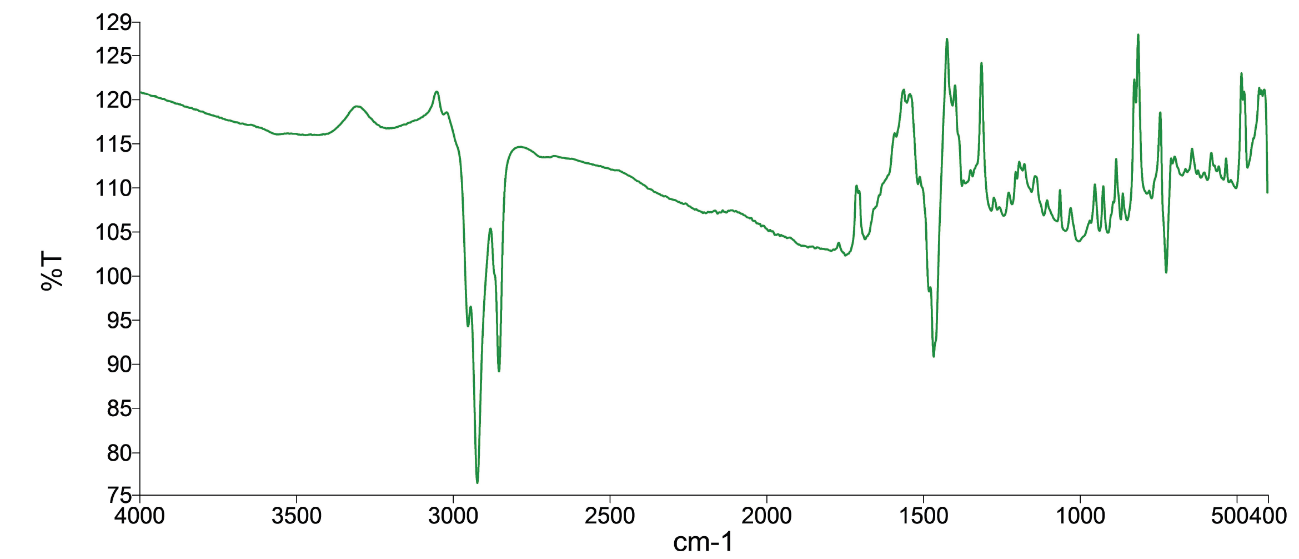


B
